# Effect of *Pistacia atlantica* subsp. kurdica Gum in Experimental Periodontitis Induced in Wistar Rats by Utilization of Osteoclastogenic Bone Markers

**DOI:** 10.3390/molecules25245819

**Published:** 2020-12-10

**Authors:** Shokhan H. Azeez, Shanaz M. Gaphor, Aram M. Sha, Balkees T. Garib

**Affiliations:** 1 Department of Dental Nursing, Sulaimani Technical Institute, Sulaimani Polytechnic University, Sulaimani 46001, Kurdistan, Iraq; 2 Department of Oral Diagnosis, College of Dentistry, University of Sulaimani, Sulaimani 46001, Kurdistan, Iraq; shanaz.gaphor@univsul.edu.iq (S.M.G.); balkees.garib@univsul.edu.iq (B.T.G.); 3 Periodontics Department, College of Dentistry, University of Sulaimani, Sulaimani 46001, Kurdistan, Iraq; aram.hamad@univsul.edu.iq

**Keywords:** bone loss, experimental periodontitis, osteoclasts, *Pistacia atlantica* subsp. kurdica, Porphyromonas gingivalis

## Abstract

The aim of this study was to assess the effect of local application of essential oil of *Pistacia atlantica* kurdica (EOK) gel in treatment of experimentally induced periodontitis in rats and its effect on osteoclastogenic bone markers. Twenty-four male Wistar rats of 250 to 350 g were used in this study and were allocated into four groups. Control negative (without induced periodontitis), control positive (induced experimental periodontitis left without treatment), treatment control (induced experimental periodontitis and treated with Chlorhexidine gel) and EOK treated group (induced experimental periodontitis treated with EOK gel). The animals were sacrificed after 30 days, and the mandibular central incisor and surrounding tissue were dissected from the mandible and further processed for preparing H&E slides. Inflammatory cells, osteoclast cells, and periodontal ligament (PDL) were examined and measured histologically. Finally, the mean concentrations of both markers, receptor activator of nuclear factor kappa-Β ligand (RANKL) and (Interleukin-1β) IL-1β, were analyzed by ELISA. A significant reduction of inflammatory reaction and osteoclast numbers with improvement of PDL and low mean concentrations of RANKL and IL-1β were seen in the EOK treated group in comparison to the control group and the chlorhexidine group as well. The extract showed a protective effect in the healing of periodontitis that had been induced in rats and decreased bone resorption by down regulation of serum RANKL and IL-1β markers.

## 1. Introduction

Periodontitis is a chronic inflammatory disease of the tooth-supporting structure. It causes inflammatory cell infiltration followed by destruction of the connective tissue, cementum, bone, and the formation of periodontal pockets [1,2]. The initiation and progression of periodontitis is connected to the presence of bacterial plaque, which enhances a local inflammatory response [3]. The inflammation causes vasodilatation, edema, infiltration by inflammatory cells and the release of a group of pro-inflammatory cytokines (including IL-1β). This process promoted an alveolar bone resorption, which could result in tooth loss [4].

Alveolar bone loss serves as a cardinal pathological and clinical feature for periodontitis. Bone is a dynamic structure that is remodeled continuously. In bone remodeling, there is a balance between bone resorption by osteoclasts and bone formation by osteoblasts. This action is regulated by the effect of receptor activators of nuclear factor-kB ligand (RANKL) and osteoprotegerin (OPG) [5]. RANKL is expressed by several cells including osteoblasts, fibroblasts, activated T cells, and B cells, which stimulates the differentiation and maturation of osteoclast precursor cells into active osteoclasts [6,7].

Many studies have been conducted to find effective drugs for the prevention and treatment of bone resorption in periodontitis. Recently, several compounds of extracted herbs and medicinal plants have been reported to exhibit anti-inflammatory, antioxidant, and antimicrobial effects. These features may suggest their potential role as pharmacological agents in modulating the host inflammatory cascade and bone resorption in treatment of periodontitis [8,9].

The mastic “Gum” exudates from the stem of *Pistacia atlantica* kurdica tree (Betoum). It was traditionally used as a remedy for the treatment of upper abdominal and gastric pain, dyspepsia, and peptic ulcer [10]. This plant’s extract was used as a wound dressing in Kurdistan, Iran, due to being potentially safe, cost effective, and efficient in wound healing [11,12]. *Pistacia* species have caught the interest of researchers and different parts of this plant, including the leaves, kernels, hulls, and gum have been studied and have demonstrated antioxidant, antimicrobial, and anti-inflammatory activities [13]. Several parts of the different plants biosynthesize essential oils, which have been commonly used for combating pathogens such as bacteria, fungi, and viruses for many years [14]. The therapeutic role of plant-derived essential oil products in the treatment of periodontitis have been widely studied [15].

For the first time in the Kurdistan region of Iraq, the antibacterial activity of essential oil of *P. atlantica* gum against a clinically isolated *p. gingivalis* in vitro model has been reported. The polyphenols and flavonoids components of *Pistacia atlantica* kurdica (EOK) are a promising agent in wound healing because of their powerful antioxidant, and anti-inflammatory effect [16]. However, no study has been conducted on the effects of essential oil extracted from *P. atlantica* gum on experimental periodontitis. This study was designed to evaluate whether the extract could control the inflammatory reaction and inhibit alveolar bone resorption in animal models of experimental periodontitis.

Interleukin-1 (IL-1) stimulates the proliferation of keratinocytes, fibroblasts, and endothelial cells of the periodontal tissues. Additionally, it enhances fibroblast to synthesis type I procollagen, collagenase, hyaluronate, fibronectin, and prostaglandin E_2_ [17]. Therefore, IL-1 is a vital component in the homeostasis of periodontal tissues. Its excessive expression could result in tissue damage [18]. Interleukin 1β (IL-1β) upregulates matrix metalloproteinase and downregulates tissue inhibitors of metalloproteinase production. It is well documented that (IL-1β) has been involved in the pathogenesis of inflammation induced bone resorption [19].

The receptor activator of nuclear factor-κB ligand (RANKL) is an essential substance in the regulation of differentiation, recruitment, and function of osteoclasts [20]. RANKL molecules are regarded as the main bone metabolism regulators and are also considered to be crucial for the mechanism of periodontal destruction in periodontitis, in which periodontal fibroblasts are induced either by mechanical forces or bacterial challenge [21]. 

Our goal was to assess the efficacy of *P. atlantica* gel in improving experimentally induced periodontitis in Wistar rats by Histopathological analysis and evaluation of RANKL and IL-1β levels ELISA. 

## 2. Results

Table 1 shows the outcomes achieved by Gas Chromatography-Mass Spectrometry (GC-MS) analyses of the components of essential oil of *P. atlantica* gum, along with their retention times, retention indices, and percentage shares. Analysis showed that 29 compounds were recognized, among which α-Pinene (79.76%) has been described as the main compound of the *P. atlantica* gum essential oil [16].

### 2.1. Histopathological Results

#### 2.1.1. Control Groups

**Figure 1:**
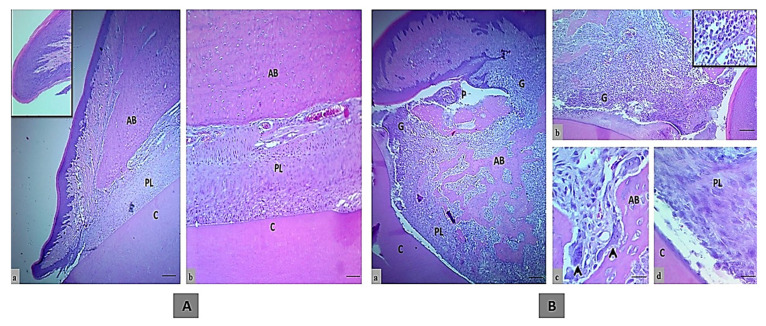
The histologic section of an incisor tooth and periodontal tissue of rats. (**A**) The control negative group, a and b. Normal histological and structural organization of the intact gingival lining epithelium and periodontal tissues (inset is normal histological features in low magnification), (H&E, scale bar 10 μm in section a and b). (**B**) Control positive group, a–c. Marked periodontal pocket (P) with disruption of the junctional epithelium and granulation tissue in the insertion point, also above the bone crest that indicated by G and inset, that showed the higher magnification of inflammatory cells infiltration, disorganized bone trabeculae (BT) and irregular bone surface with a presence of osteoclasts involved in bone matrix cavity (black arrows), d. The wide periodontal ligament space is filled with disorganized fiber and proliferating periodontal ligament tissue that has not attached to the cementum, (H&E, scale bar 10 μm in section a, and 20 μm in section b and 40 μm in section c and d). (AB; alveolar bone, PL; periodontal ligament and C; cementum).

The histology of an incisor tooth and periodontal tissues of rats in the control negative group sacrificed after one month, comprising gingival tissue, periodontal ligament, and alveolar bone, displayed normal structures and arrangement without any indication of inflammation or bone loss (Figure 1A).

Meanwhile, the main histologic feature of the periodontal tissue of rats in the control positive group showed severe damage of the periodontal tissue in comparison to the rats in the control negative group. This damage included disruption of the gingival epithelial lining tissue that led to pocket formation and severe infiltration into the insertion point of inflammatory cells such as neutrophil and mononuclear cells (macrophages, plasma cells, and lymphocytes) that were found in gingival epithelial tissue, periodontal ligament and alveolar bone, even in granulation tissue in the crest, and also in the alveolar bone, as seen in Figure 1B.

Active bone resorption was found along with high numbers of osteoclasts involved in a large bone lacuna resorption (Howship’s lacuna) with ruffled border, marking the irregular bone surface. The newly formed bone trabeculae were disorganized. There was a newly formed layer of osteoid tissue that contained a large number of osteoclast, fibroblast, mononuclear inflammatory cells and dilated blood vessels. The periodontal ligament was partially degenerated and showed disorganized proliferation of periodontal ligament tissue (collagen fibers and fibroblast) that had not attached to the cementum surface.

#### 2.1.2. Treatment Groups

**Figure 2:**
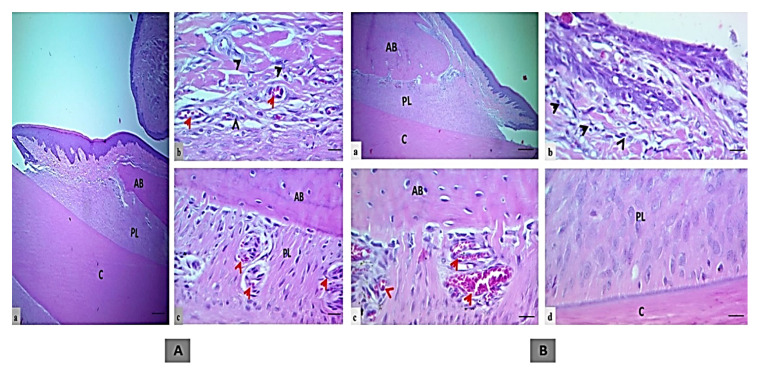
The histologic section of an incisor tooth and periodontal tissue of rats. (**A**) Treatment control group, a and b. Intact junctional epithelium with mild inflammatory cells (black head arrows) in the insertion point, c. Regular bone surface with well-formed dense bone, a wide periodontal ligament space of uniform thickness filled with organized proliferating periodontal ligament tissue attached to a regular cementum surface and multiple blood vessels (red arrow, H&E, scale bar 10 μm in section a, and 20 μm in section b and c). (**B**) EOK or tested treatment group, a and b. Intact junctional epithelium with mild inflammatory cells (black head arrows) in the insertion point (section b), c. Regular bone surface with well-formed dense bone, and dilated blood vessels (red arrow), d. A wide periodontal ligament space of uniform thickness filled with less organized proliferating periodontal ligament tissue attached to a regular cementum surface (H&E, scale bar 10 μm in section a, and 20 μm in section b–d). (AB; alveolar bone, PL; periodontal ligament and C; cementum).

Microscopical sections of an incisor tooth and periodontal tissues in the treatment control group showed intact junctional epithelium with a mild inflammatory reaction in the insertion point. The bone surface was regular in appearance with well-formed dense bone. Periodontal ligament space was wide and with uniform thickness that was filled with organized proliferating periodontal ligament tissue attached to a regular cementum surface, as shown in Figure 2A.

However, the EOK treatment group also exhibited intact junctional epithelium with mild inflammatory cell infiltration in the insertion point with regular bone surface and well-formed dense bone. In comparison to the treatment control group, a wide periodontal ligament space of uniform thickness filled with less organized proliferating periodontal ligament tissue attached to a regular cementum surface was found, as shown in Figure 2B.

### 2.2. Effect of Essential Oil of Pistacia atlantica on Inflammatory Cells in Experimental Periodontitis

The statistical analysis of the polymorphonuclear and mononuclear inflammatory cells at 30 days among the different groups is shown in Table 2. The result showed an increase in inflammatory reaction in the control positive group by score 2 in comparison to the control negative group (score 0), which led to severe damage of periodontal tissue. The mean of the polymorphonuclear inflammatory cells was (25.6 ± 5.79), the highest cellular infiltration among these was of neutrophils with high significant value (*p* = 0.005), followed by eosinophils with significant value (*p* = 0.02) and non-significant infiltration of basophils (*p* = 0.18). Meanwhile, the mononuclear inflammatory cells showed highly significant infiltration with mean (33.9 ± 10.38), and increased significance of the plasma and macrophages cells (*p* = 0.02 and 0.03), in contrast to the treatment control group, where all types of inflammatory cells decreased (score 1); for instance, the mean of the polymorphonuclear inflammatory cells was (18.2 ± 3.88), with the neutrophils having significantly declined (*p* = 0.004), followed by eosinophils and basophils at *p* = 0.16 and 0.13, respectively, while the mononuclear inflammatory cells had fallen significantly to (17.2 ± 4.38), with the plasma and macrophages cells showing a significant effect (*p* = 0.03 and 0.02). Interestingly, both types of inflammatory cells in the EOK treated group had decreased by score 1, for the polymorphonuclear cells (8.7 ± 1.61), with *p* = 0.06 for the neutrophils and a non-significant reduction (*p* = 0.34 and 0.65) of eosinophils and basophils, respectively. In contrast, the mononuclear inflammatory cells had slightly increased (15.4 ± 21.43), with *p* = 0.13 recorded for the plasma cells and 0.39 for the macrophages, which specified a significant effect of the P. atlantica oil in decreasing the inflammatory cells. In comparison to the control treatment group, a significant decrease in inflammatory reaction was seen in the rats treated with EOK (*p* = 0.02).

### 2.3. Effect of Essential Oil of Pistacia atlantica on the Thickness of Periodontal Ligament in Experimental Periodontitis

**Figure 3:**
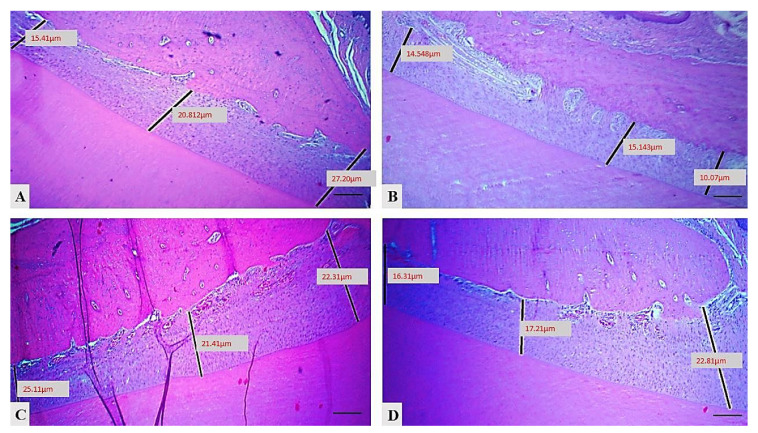
Variations in periodontal ligament thickness across the different groups: (**A**) control negative group, (**B**) control positive group, (**C**) treatment control group, (**D**) EOK treatment group, (H&E, scale bar 10 μm).

Figure 3 and Table 3 show a significant decrease in value (30.43 ± 1.30, *p* = 0.08) in periodontal ligament thickness in the control positive group in comparison to the control negative group (18.90 ± 1.01), with an increase in the occupation of space by inflammatory reaction and tissue damage. In the treatment group the space slightly decreased and there was significant improvement in the thickness of the PDL; for instance, the mean (21.38 ± 1.99) with *p* = 0.09 in the treatment control and the EOK treated group showed marked improvement in PDL thickness (21.76 ± 0.87, *p* = 0.02) in comparison to the control positive group.

### 2.4. Effect of Essential Oil of Pistacia atlantica on Osteoclast Numbers in Experimental Periodontitis

**Figure 4:**
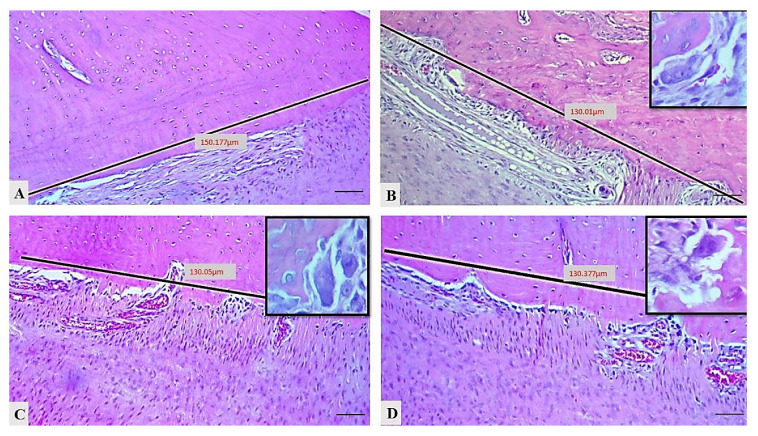
Histometric counting of osteoclasts (insets showed multinucleated, ruffle border osteoclasts) in diverse groups: (**A**) control negative group, (**B**) control positive group, (**C**) treatment control group, (**D**) EOK treatment group, (H&E, scale bar 20 μm).

The mean numbers of osteoclasts in the different groups in this experiment, as displayed in Table 4 and Figure 4, indicate that the number of osteoclasts significantly rose (4.33 ± 0.84, *p* = 0.004) in the control positive group in comparison to the control negative group. Interestingly, the number of osteoclasts had reduced in both treated groups in comparison to the control positive group; for example, the mean number had dropped significantly in the control treatment group (1.16 ± 0.47, *p* = 0.4), while in the EOK treated group it decreased with a highly significant effect (1.33 ± 0.42, *p* = 0.009).

### 2.5. Assessment of Effect of Essential Oil of Pistacia atlantica on IL-1β Concentration by ELISA

Table 4 illustrates the evaluation of IL-1β mean concentrations in the different groups. IL-1β levels in the control positive group were significantly higher than in the control negative group and reached 8666.66 ± 299.62 pg/µL by *p* = 0.000. Whereas IL-1β concentration decreased significantly in the treatment groups in comparison to the control positive group; in particular, the group treated with *P. atlantica* revealed a significant difference (*p* = 0.000) and about 117-folds reduction in the IL-1β concentration compared to the treatment control group’s result of 4400.00 ± 288.67 pg/µL with *p* = 0.01.

### 2.6. ELISA Evaluation of Effect of Essential Oil of Pistacia atlantica on RANKL Concentration

Table 4 presents the mean concentrations of RANKL in the studied groups. As shown, there was a highly significant (*p* = 0.000) increase in the concentration of RANKL in comparison to the control negative group (514.16 ± 44.35 pg/µL). Meanwhile, there was a highly significant (*p* = 0.02) reduction in the concentration of the RANKL in the EOK treated (301.66 ± 29.59 pg/µL) group in comparison to the control positive group, and even compared to the treatment control group which recorded (343.33 ± 21.55 pg/µL) with *p* = 0.56.

## 3. Discussion

Periodontal diseases are characterized by chronic inflammatory lesions initiated and propagated by the accumulation of sub gingival biofilm that occurs due to the inflammatory reaction of the immune response of the host against periodontal pathogens. Resorption of alveolar bone, pocket formation, and inflammation are features of periodontitis [22]. The discovery of a new therapy that may inhibit or decrease bone loss creates the opportunity to find a drug that will act not only on the inflammatory reaction but also affect the destruction of bone loss that happens in periodontitis.

Our histological findings showed that the extract of essential oil of *P. atlantica* gum improved the damage associated with ligation and *Porphyromonas gingivalis* injection and also decreased loss of alveolar bone, at the same time improving the PDL thickness about 8.67 folds compared to the control positive group that exhibited marked alveolar bone loss and disruption of the periodontal tissues, and marked damage to the PDL of about (30.43 ± 1.30). Moreover, the *P. atlantica* had a better or the same effect as the chlorhexidine, which is conventionally used for treatment of periodontitis and, according to former reports, is effective in a topical application of 0.2% chlorhexidine gel as a periodontal therapy through the reduction of pellicle formation, modification of bacterial adherence to teeth, and lysis of bacterial cell walls [23,24,25]. In the current study the improving effect of *P. atlantica* oil was due to the presence of antioxidant components such as phenols and flavonoids compounds that are effective in lipid peroxidation reduction and can enhance vascularity, collagen formation (which is the main component of the PDL), and increase cross linking of collagen fibers, as documented by a former study [26].

The presence of bacteria at sites of periodontitis may cause a delay in the starting of the proliferative and remodeling stages of wound healing phases due to the releasing of free radicals and lytic enzymes that also cause disruption and damage of the periodontal ligaments and tissues [27]. The decreasing of the bacterial load in periodontal tissue in the EOK treated group in comparison to the control positive group is due to antibacterial activities of this plant that stimulate healing by acceleration of wound contraction and re-epithelialization of the tissue, and our finding confirms a previous report, which documented that the presence of high concentrations of alpha-pinene in the composition of the oil causes disruption of bacterial cell membrane integrity [28], indicating the bactericidal activity of this extract on wound healing and its attenuation of the severity of periodontal tissue abnormality. 

In our results, the inflammatory reactions or infiltration after 30 days were decreased in the *P. atlantica* oil treated groups (score 1) in comparison to the control positive group (score 2) and the treatment control group (score 1). This outcome is in accordance with a previous study’s finding of an anti-inflammatory effect of our extract oil that may be related to the presence of oleanonic acid, which reduces the production of leukotriene B4 [29]. Additionally, it is in agreement with a recent study that demonstrated the anti-inflammatory effectivity of our product by decreasing the numbers of both polymorphonuclear and mononuclear inflammatory cells [16].

IL-1β is a multifactorial cytokine that is able to activate many cell types with potent inflammatory features. The wide biological effects of IL-1β play a central role in the regulation of many different genes. IL-1β affects approximately 90 genes that occur during inflammation that spread in the gingival connective tissue and lead to the occurrence of attachment loss and alveolar bone destruction due to its enhanced effect on osteoclastogenesis [30]. 

Plasma interleukin analysis in our study illustrated that the amount of IL-1β was much higher in rats’ blood in the control positive group than in healthy rats. Therefore, this group displayed severe destruction of PDL ligaments and connective tissue with marked periodontitis in comparison to the treatment groups; also, our finding is in agreement with authors who demonstrated that aggressive bone destruction correlated with the presence of high levels of proinflammatory IL-1β in periodontal disease [31,32], which is in accordance with our finding that high mean concentration of IL-1β is related to marked bone loss. Additionally, Feghali et al. mentioned that IL-1β stimulates production and extracellular release of high mobility group box 1 protein (HMGB1) from murine fibroblast, Apoptotic and necrotic cell deaths of gingival fibroblasts resulted in the enhancement of HMGB1 [33].

Furthermore, in the treatment groups, and more specifically in the EOK treated group, the mean concentration of the IL-1β decreased more significantly than in the control positive group and even the group treated with chlorhexidine; therefore, the periodontal tissue of this group exhibited less PDL destruction and milder periodontitis due to the anti-inflammatory and antioxidant effects of *P. atlantica* [16] in reducing oxidative damage and suppressing inflammatory mediators.

Our outcomes indicated that the progression of periodontal disease was more significantly associated with increased levels of RANKL in the control positive group than in the control negative group, possibly leading to marked bone loss and elevation of osteoclast numbers. As demonstrated previously by many studies, RANKL is a positive marker of bone resorption and production of RANKL modulates osteoclastogenesis and bone remodeling. This would explain the bone loss in periodontitis and it indicates that RANKL could play an important role in periodontal bone resorption and its inhibition might decrease resorption of periodontal bone [34].

Interestingly, the current study demonstrated that mean concentrations of RANKL were lower in the experimental periodontitis rats treated with the *P. atlantica* gum oil when compared with the control positive group and control treated group. There was also a reduction in alveolar bone loss through a decrease of the osteoclast regulators (RANK) gene. Additionally, our study is in agreement with studies which reported that upregulated RANKL levels are related to the number of *P. gingavalis.* A lysine-specific gingipain produced by the periodontal pathogen *P. gingivalis* are able to degrade cytokine and has been reported to cause pathological bone destruction by degrading osteoprotegerin, and a decoy receptor of RANKL [35,36]. This could explain why in the EOK treated group the level of this gene was decreased due to the bactericidal effect of this product [26]. This finding also shows that RANKL plays an important role in periodontal resorption and as well as RANKL suppression can reduce periodontal bone resorption and bone loss.

Another explanation for the *P. atlantica* gum oil decreasing the number of osteoclasts is that this extract contains a phenolic compound [21], which is responsible for osteoclast genesis suppression [25].

## 4. Materials and Methods

### 4.1. Essential Oil Isolation, Gas Chromotography-Mass Spectrometer (GC-MS) Analysis, and Identification of Constituents

The gum collection of *P. atlantica* (pistachio tree of the Atlas) was done in June and July 2017 from the Halabja region, which is 70 km far from Sulaimani city in the north of Iraq. The essential oil was obtained from the gum using the hydrodistillation method; 100 g of gum was soaked in 350 mL of distilled water in a conical flask and left for 6 h using the Clevenger apparatus according to [37,38]. Next, the essential oil was collected after 6 h of extraction. 

The chemical composition of essential oil of *P. atlantica* was determined by GC-MS method. The sample was run on the Shimadzu QP-2010 GC-MS (Shimadzu, Kyoto, Japan) method with a non-polar columnDB-5 (30 m × 0.25 mm × 0.25 μm) which was linked directly with the MS. The oven temperature was set to 40 °C for 2 min and then held isothermally at 280 °C for 2 min, while the injector port was held at 280 °C. Essential oil (1 μL) with hexane (1:1) was injected and the split ratio was 1:5. Data capture took place at 70 eV using 1.5 s scanning times in the 50 to 800 amu mass range. Mass spectra and chromatography were handled with Chem station software Shimadzu GC-MS solution ver.4 software (Tokyo, Japan). 

The individual peaks/constituents were identified by comparison of their Kovats Index (K.I.) either with those of authentic compounds available in the author’s laboratory or with those of literature in close agreement to K.I. Further identification of essential oil constituents was made by comparison of the fragmentation pattern of mass spectra obtained by GC-MS analysis with those stored in the spectrometer database of NIST, NBS 54 K.L, WILEY8 libraries, and published literature [39,40,41,42].

### 4.2. Animal Model

The 24 male Wistar rats of 250 to 350 g used in this study were purchased from the animal house of the College of Veterinary Medicine, University of Sulaimani. They were then housed under a 12/12 h light/dark cycle at a temperature of 22 ± 2 °C with water and food ad libitum. The principles of handling animals were according to the institutional guidelines and all experiments were performed in accordance with the Ethical Committee for Animal Research of Sulaimani University (112670).

### 4.3. Experimental Design

**Figure 5:**
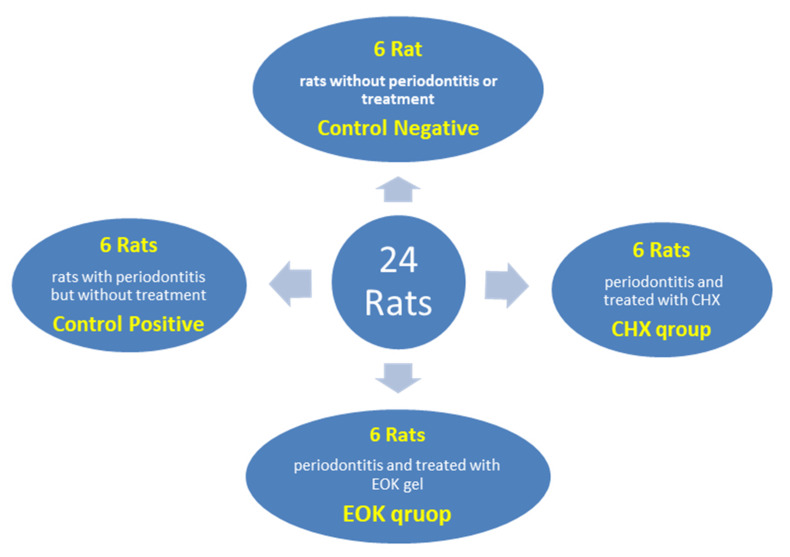
Experimental design of the studied groups.

The 24 Wistar rats were randomly divided into four groups with six rats in each group, as shown in Figure 5:

Control negative: rats were not inoculated with the *Porphyromonas gingivalis* + ligature and not treated with any agent.

Control positive: rats were injected with the *P. gingivalis* + ligature and left without treatment.

Treatment control: rats were injected with the *P. gingivalis* + ligature and then treated with Chlorhexidine 0.2% gel.

EOK treated group: rats were injected with the *P. gingivalis* + ligature and then treated with (*P. atlantica*) gel 12.5 µL/mL. 

A pre-experimental examination was done to ensure that animals were periodontal disease-free before the induction of disease, with periodontal probing depths not exceeding 0.5 mm (Chicago probe, IL, USA) [43]. 

### 4.4. Induction of Experimental Periodontitis

Experimental periodontitis was induced by a combination of ligature and application of *P. gingivalis* as follows: after anesthetizing the animals, the necks of the cervix of both mandibular central incisors were ligated with a sterilized black braided nylon thread No 4-0 (Surgilon; USS/DG, Norwalk, CT, USA) sutures. As previously described, this ligature induced gingival irritants and promoted the accumulation of plaque and, subsequently, the development of periodontal disease and, checked daily [44], they were subsequently injected with prepared clinically isolated strains of *Porphyromonas gingivalis* which were grown in nutrient broth supplemented with 5 µg/mL hemin (Sigma Aldrich, Shanghai, China), 1 µg/mL vitamin K1 (HiMedia, Mumbai, India), and incubated using an anaerobic jar and anaerobic gas packs (AnearoPack system; Mitsubishi Gas Chemical, Tokyo, Japan) for about 2 days. 

*P. gingivalis* turbidity of (0.8) optical density was determined spectrophotometrically at 600 nm, using (0.03 mL) of a mixture of bacteria and 4% sodium carboxymethyl cellulose (PanReac Química SLU, Barcelona, Spain) with a concentration of (2 × 10^6^ CFU/mL). Before injecting the animals with *P. gingivalis,* they were anesthetized with a combination of a mixture of ketamine hydrochloride (3.3 mL) and xylazine hydrochloride (2 mL) by intraperitoneal injection at a dose of 0.05 mL/Kg/BW, then *P. gingivalis* was locally injected into the gingival sulcus at the bottom of the lower incisor teeth and this procedure was performed three times per week for two weeks [45].

### 4.5. Gel Preparation

The formulation and preparation of Muco-adhesive gels of CHX and EOK were done by the (Awa Medica Drug Company, Hawler, Iraq) using a simple dispersion method.

#### Method of Preparation

**Essential Oil of *Pistacia atlantica* kurdica (EOK) Gel:** Gel prepared by dissolving 4 mg of Metalose 90SH 10000 (Shin-Etsu Chemical Co., Tokyo, Japan) in 850 mL purified water; the solution was mixed with 100 mL Propylene glycol (Pharmaco-AAPER, Karnatka, India) Using tissue homogenizer. (12.5 µL/mL of essential *P. Atlantica*) was transferred into solution and homogenized, 25.00 mg of potassium sorbate (Analtik kimya ve lab., Istanbul, Turkey) was added. Then, distilled water was added to make final volume to 1000 mL, mixing and homogenizing them continuously until a transparent homogenous gel is formed. The gel was stored at ambient temperature.

**Chlorohexidine (CHX) Gel:** Control gel of CHX was prepared in the same manner as above by replacing EOK with % 0.2 of Chlorohexidine digluconate (HiMedia). 

### 4.6. Treatment of Experimental Periodontitis

The gel was administered once a day via local application of materials (Chlorhexidine 0.2% gel in CHX treatment group and EOK gel in EOK tested group) through a disposable plastic syringes with a needle having a blunt end of gauge (22) were used to transport the materials into the nearby gingival cervix at the bottom of the lower incisor teeth for 30 days (i.e., from day 0 to day 30). The rats were evaluated daily throughout the experiment to check the loosening sutures and for clinical or toxicological symptoms.

### 4.7. Histological Assessment

After the 30 days of the experiment, all animals were sacrificed, and the right mandibular central incisor was removed from the mandible, fixed in 10% buffered neutral formalin for 2 days, decalcified in a 10% EDTA solution for 6 weeks at 4 °C and further processed for preparing H & E slides. 

**Figure 6:**
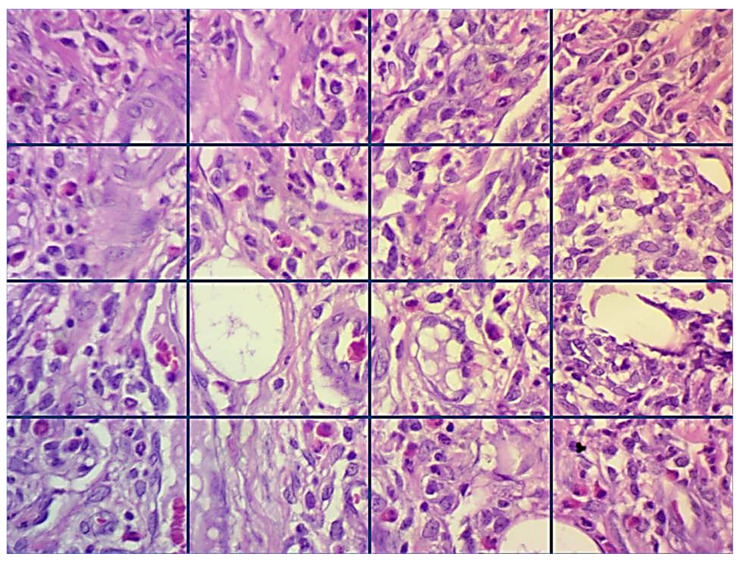
Scheming a method for inflammatory cells counting (H&E stains, 400×).

Inflammatory cells were quantitatively evaluated at the site of damage after inducing periodontitis, whether treated or not treated, in 2 randomly selected fields at 400× by applying a light microscope (Leica Motic, Hong Kong, China), connected with an image analyzer software (AmScope, Tokyo, Japan, 86×, 3.7.4183, 2014). For each area, a picture was captured and then partitioned into 16 squares (Figure 6), then all inflammatory cells (polymorphonuclear cells and mononuclear cells) were counted and an average number for each group was achieved. The inflammatory cells were scored and classified as follows: score 0 (0–25 inflammatory cells), mild or score 1 (26–50 inflammatory cells), score 2 (51–75 inflammatory cells), and score 3 (more than 75 inflammatory cells) [16].

The thickness of the periodontal ligament (PDL) was measured in three positions (crest, middle, and cervical portion) perpendicularly from the alveolar bone surface to the cementum or cementum surface using histometric analyzer software (Amscope^TM^, Tokyo, Japan), then the mean was taken from each of the three mentioned layers in each case for each group.

The number of osteoclasts in the tissue sections was counted in 130 µm by histometric analyzer software (Amscope^TM^, Tokyo, Japan) from the alveolar bone surface in each rat in the groups and detected in H&E stain sections as multiple nuclei, ruffled border, and granular cytoplasm. Finally, statistical analysis was conducted for the osteoclast counts and the PDL thickness.

### 4.8. Enzyme-Linked Immunosorbent Assay (ELISA) for the RANKL and IL-1β

Blood was drawn from each rat by intracardiac puncture just before they were sacrificed. The blood samples were transferred into tubes, centrifuged at 3000× *g* for 10 min, and dissociated to serum. The acquired serum samples were kept in tubes at −80 °C until analysis. IL 1β and RANKL (E0119Ra and E0289Ra respectively; Bioassay Technology Laboratory, Shanghai, China) levels were analyzed using ELISA kits in accordance with the manufacturer’s instructions.

### 4.9. Measurement of Alveolar Bone Loss

After scarifying of the rats, the fixed samples were fixed perpendicularly on heavy body impression materials to take a radiograph of them using CBCT (cone bean computed tomography) and a scanner Model-Cat (Imaging Science International) with an exposition area of 6 cm^2^ and exposition time of 40 s, and of voxel of 0.2 mm (maximum resolution). The digital images were analyzed using their axial, sagittal, and coronal cuts in the program i-Cat Cone Beam 3D Dental Imaging System, Vesion 3.1.62, Verona, Italy. The horizontal bone loss was determined by measuring the distance the cusp tip and alveolar bone in the long axis of the lower incisor in lingual and mesial side, in units of mm/rat, in a modification method used by Crawford et al. [46]. 

### 4.10. Statistical Analysis

A statistical analysis was performed using software package, SPSS (version 22.0, Chicago, IL, USA). The statistical analysis of variables between the experimental groups was done using one-way ANOVA. Tukey’s test was done to make a pairwise comparison between groups, also, the paired sample t-test was applied to the dependent variables to obtain separate averages for the polymorphonuclear and mononuclear inflammatory cells among the groups. Values were presented as mean ± standard error (SE) with *p <* 0.05 regarded as statistically significant.

## 5. Conclusions

*Pistacia atlantica* kurdica gel has shown a significant reduction in gingival inflammation and pocket depth, therefore demonstrating a good anti-inflammatory effect in this study. Here, for the first time, we confirmed a protective effect of *P. atlantica* kurdica gel against bone loss in induced periodontitis by the down regulation of RANKL and IL-1β markers through ELISA analysis. However, further studies and with other cytokines are required in order to demonstrate the inhibitory effect of *Pistacia atlantica* on the plasma levels of these cytokines. Hence, this extract can be used as an useful adjunct to enhance the outcomes of standard periodontal therapy.

## Figures and Tables

**Table 1:** GC Gas Chromatography-Mass Spectrometry (GC-MS) analysis of essential oil of *Pistacia atlantica* kurdica.

Peak No.	Retention Time	Kovats Index	Area %	Chemical Compounds
1	4.641	926	0.63	Tricyclene
2	4.938	939	79.76	α-Pinene
3	5.034	953	2.57	Camphene
4	5.292	977	1.31	Sabinene
5	5.357	980	4.61	β-Pinene
6	5.490	990	1.07	β-Myrcene
7	5.613	1005	0.16	α-Phellandrene
8	5.658	1010	0.12	2-Carene
9	5.727	1022	0.73	3-Carene
10	5.821	1029	0.12	*p*-Cymene
11	5.914	1026	0.40	α-Terpinene
12	5.973	1039	2.13	*D*-Limonene
13	6.309	1062	0.07	γ-Terpinene
14	6.626	1091	1.16	γ-Terpinolene
15	6.772	1098	0.09	Linalool
16	7.081	1110	0.20	α-Camphenol
17	7.256	1139	0.34	*trans*-Pinocarveol
18	7.301	1153	0.45	Pinocarvone
19	7.392	1165	0.09	Borneol
20	7.568	1177	0.09	Terpinen-4-ol
21	7.660	1285	0.17	Bornyl acetate
22	7.737	1295	1.17	Thymol
23	7.825	1305	0.17	Carvacrol
24	8.684	1400	0.84	Tetradecane
25	8.844	1471	0.08	Acetic acid
26	9.000	2000	1.04	Eicosane
27	9.714	2649	0.31	Phthalic acid, bis(7-methyl octyl) ester

**Table 2:** The mean ± SE of inflammatory cells in the different groups.

Groups	Inflammatory Cells Mean ± SE (Total)	Polymorph Nuclear Inflammatory Cells Mean ± SE			Mononuclear Inflammatory Cells Mean ± SE			
		Neutrophil	Eosinophil	Basophil	Lymphocyte	Plasma cell	Macrophage	
Control Negative	12.2 ± 2.26	3.10 ± 0.54	0.00 ± 0.00	2.50 ± 0.54	0.00 ± 0.00	2.00 ± 0.29	4.60 ± 0.89	
Control Positive	59.5 ± 16.17	20.20 ± 4.35 ***	0.60 ± 0.22 ***	4.80 ± 1.22	0.00 ± 0.00	16.50 ± 5.02 ***	17.40 ± 5.36 ***	
Treatment Control (Chx)	35.4 ± 8.26	13.10 ± 2.64 ***	0.20 ± 0.13	4.90 ± 1.11	0.00 ± 0.00	7.40 ± 2.13 ***	9.80 ± 2.25 ***	
Test (EOk) Treatment	24.1 ± 7.64	6.50 ± 1.01 ***	0.10 ± 0.10	2.10 ± 0.50	0.00 ± 0.00	8.50 ± 3.93	6.90 ± 2.10	

Within each row values are expressed as mean ± SE values, *** *p* < 0.01 vs. control (*n* = 6).

**Table 3:** Analysis results for PDL thickness in each of the study groups.

Control Negative (No = 6)	Control Positive (No = 6)	Treatment Control (No = 6)	EOK Treatment (No = 6)
15.78	31.40	18.60	18.98
20.54	29.50	24.49	23.76
17.15	26.92	23.68	23.31
21.60	26.97	27.55	23.17
21.18	34.64	13.80	22.20
17.17	33.17	20.17	19.17
18.90 ± 1.01	30.43 ± 1.30 ***	21.38 ± 1.99 ***	21.76 ± 0.87 ***

Within each row, values are expressed as mean ± SE values, with differences among them shown in the accompanying small stars; *** *p* < 0.05, vs. control (*n* = 6).

**Table 4:** Mean values for osteoclast cells, alveolar bone loss, level of IL1 β and RANK expression in each group in the study.

Groups	Osteoclast Cell Count	Alveolar Bone Loss	IL1 β (pg/µL)	RANKL (pg/µL)
Control negative	0.000	6.21 ± 0.08	1543.33 ± 194.21	203.33 ± 21.08
Control positive	4.33 ± 0.84 ***	8.86 ± 0.83 ***	8666.66 ± 299.62 ***	514.16 ± 44.35 ***
CHX treated	1.16 ± 0.47 ***	7.41 ± 0.22 ***	4400.00 ± 288.67 **	343.33 ± 21.55 **
EOK treated	1.33 ± 0.42 ***	7.15 ± 0.37 ***	4283.33 ± 197.34 ***	301.66 ± 29.59 **

Within each row, the values are expressed as mean ± SE values, with differences among them shown in the accompanying small stars: *** *p* = 0.000, ** *p* < 0.05 vs. control no = 6 in each group.
